# Mechanisms of Kaempferol in the treatment of diabetes: A comprehensive and latest review

**DOI:** 10.3389/fendo.2022.990299

**Published:** 2022-09-07

**Authors:** Yan Yang, Zhengtao Chen, Xiaoyan Zhao, Hongyan Xie, Lian Du, Hong Gao, Chunguang Xie

**Affiliations:** ^1^ Hospital of Chengdu, University of Traditional Chinese Medicine, Chengdu, China; ^2^ School of Basic Medical Sciences, Chengdu University of Traditional Chinese Medicine, Chengdu, China

**Keywords:** kaempferol, diabetes, diabetes complications, obesity, mechanism

## Abstract

Obesity–insulin resistance–β-cells apoptosis” is an important trilogy of the pathogenesis of type 2 diabetes. With the global pandemic of obesity and diabetes, continuous research and development of new drugs focuses on the prevention of the pathological progress of these diseases. According to a recent study, the natural product kaempferol has excellent antidiabetic effects. Therefore, this review comprehensively summarized the frontier studies and pharmacological mechanisms of kaempferol in the treatment of diabetes. The successful research and development of kaempferol may yield a significant leap in the treatment of diabetes and its complications.

## Introduction

Diabetes is a serious global public health concern. Approximately 451 million people were diagnosed with diabetes in 2017, and 693 million people are predicted to be diagnosed with diabetes by 2045 (1). Moreover, 374 million people have impaired glucose tolerance (1). The prevalence of diabetes varies slightly across countries and regions. It affects nearly 25.8 million people in the United States, accounting for 8.3% of the total population (2). The morbidity rate of diabetes is 11.2% among Chinese adults (3). The incidence rate of diabetes in obese and overweight individuals has increased significantly (4). The development of new antidiabetic drugs, including sodium-glucose cotransporter 2 inhibitors, has improved the survival rate of patients with diabetes. Unfortunately, the morbidiry associated with diabetes continues to increase. Therefore, the continuous development of new antidiabetic drugs is inevitable (5–8).

The prevalence of diabetes is associated with an increase in obesity (9). According to the World Health Organization, more than 1.9 billion adults worldwide are overweight, and more than 600 million people are obese (10). Excess adipose tissues in obesity release nonesterified fatty acids (NEFAs), glycerol, adipokines, and pro-inflammatory cytokines (tumor necrosis factor-α) [TNF-α], interleukin [IL]-6, IL-1β), leading to insulin resistance (IR) and type 2 diabetes mellitus (T2DM) (11). Lipid metabolism disorders, chronic inflammation and IR caused by obesity are the core pathogeneses of T2DM. Chronic exposure to NEFAs is associated with impaired glucose-stimulated insulin secretion and decreased insulin biosynthesis (12). Increased NEFA and glucose levels can occur simultaneously, and when combined, these two are significantly detrimental and lead to “glycolipid toxicity” (13). IR acts as a bridge between obesity and T2DM. Some insulin-resistant individuals maintain normal blood glucose levels. This is because islet β cells overcome the decrease in insulin efficiency by increasing insulin release (14, 15). β cells play an important role in the pathogenesis of type 2 diabetes. Expectedly, β-cell dysfunction exists in insulin-resistant individuals with normal blood glucose levels (16). In patients with type 2 diabetes, the number of β cells decreases by approximately 50%, and only 25% or less of β cells can function (17). Therefore, “obesity–IR–β-cell apoptosis” results in diabetes and its complications.

The natural product kaempferol, extracted from plants, has become the focus of studies. In recent years, natural products have accounted for 30% of global clinical drugs, and more than 65% of the global population uses natural products to treat diseases (18, 19). The development of natural products for the treatment of diabetes has attracted considerable attention (20, 21). The use of natural plants such as Ginkgo biloba, galangal, and Pueraria, has a long history, especially in Asia.

Kaempferol (3,5,7-trihydroxy-2-[4-hydroxyphenyl]-4H-1-benzopyran-4-one) is a natural flavonoids compounds with a low molecular weight (286.2 g/mol) (22). It can be found in traditional medicines, such as Sophora japonica, ginkgo, and galangal, and in foods, such as beans, cauliflower, cabbage, gooseberry, grapes, cabbage, strawberries, tea, and tomatoes (22). Kaempferol has anti-inflammatory (23), anti-oxidative stress (24), antitumor (25), anti-atherosclerotic (26), hypoglycemic (27), and hypolipidemic (28) effects.

**Figure 1:**
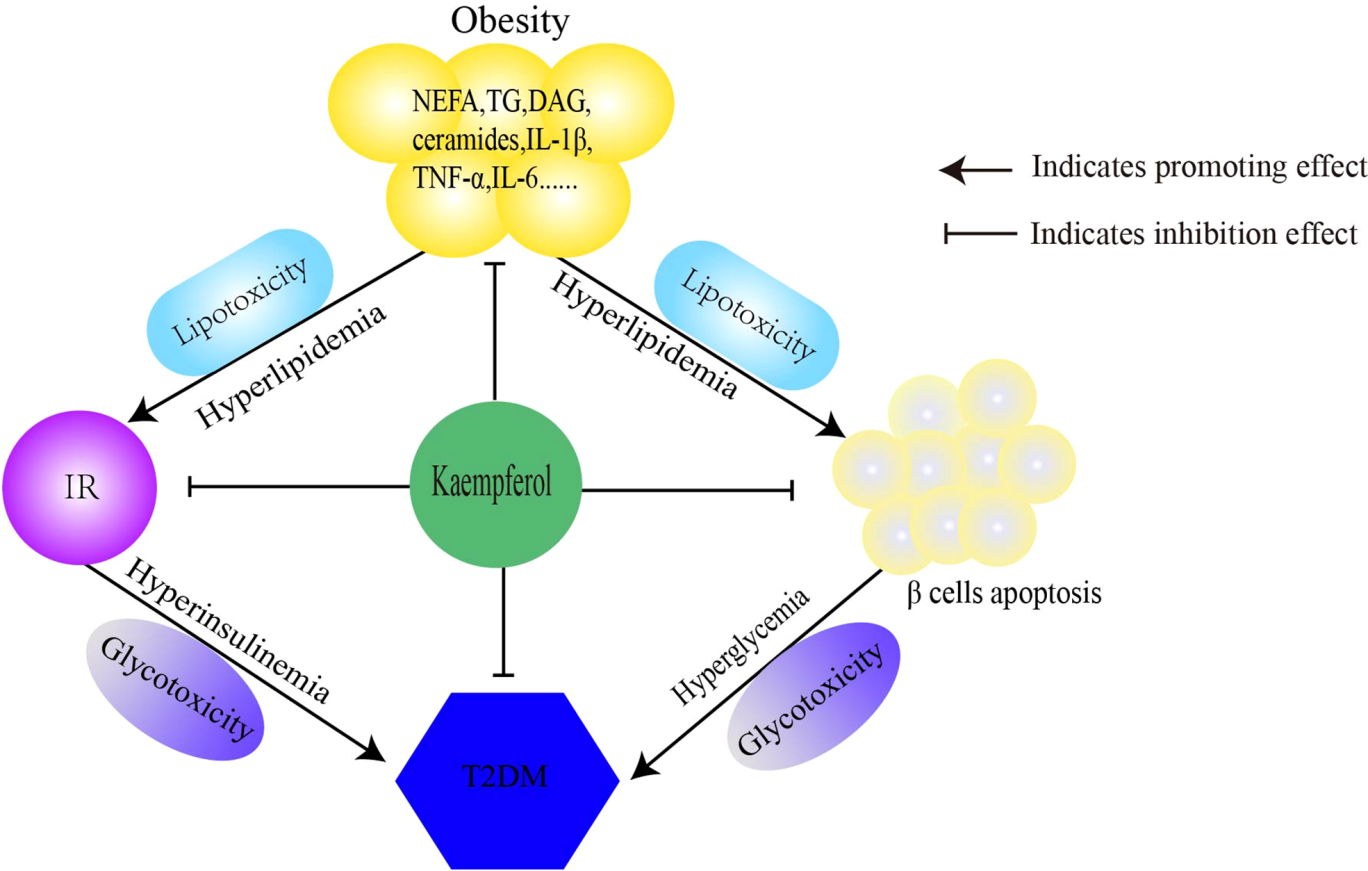
Mechanism of kaempferol antidiabetes. Kaempferol prevens the pathological progress of obesity-insulin resistance-β Cells apoptosis-diabetes. IR, insulin resistance; T2DM, type 2 diabetes mellitus.

In this article, we discussed the antidiabetic mechanisms of Kaempferol **Figure 1** from three perspectives. Kaempferol regulates lipid metabolism and improves IR to reduce lipotoxicity. Second, kaempferol improves insulin signaling and restores the balance between glucose utilization and production, thereby improving glucose toxicity. Finally, kaempferol restores the imbalance in autophagy-apoptosis to protect β cells. Therefore, the antidiabetic mechanisms of kaempferol is to comprehensively prevent the progression of “obesity–IR–β-cell apoptosis–diabetes–diabetic complications” (**Table 1**).

**Table 1:** Mechanism of kaempferol anti-diabetes.

Experimental Object	Kaempferol Dosage	Modes of Antidiabetic Action	Related Signaling Pathways or Targetes	References
3T3-L1 cells ; zebrafish	3T3-L1 cells: 7.5/15/30μM zebrafish : 5/10/20μM	(a) fatty acid synthesis↓; (b) triglyceride synthase↓;	PPARγ, C/EBP-α And ap2↓ LPAATθ, lipin1 DGAT1↓ FASN and SREBP-1C↓	29
3T3-L1 cells ;	5μM, 40μM;	(a) Lipid Accumulation↓; (b) Fatty Acid Oxidation↑;	PPARγ, LXR-a, SREBP-1c and C / EBPA↓	30
Male Wistar rats	75, 150 or 300 mg / kg	(a) fatty acid oxidation↑;	SREBPs ↓ iPPAR -α↑	31
Human mesenchymal stem cells (hMSCs)	1μM, 10μM and 25μM	(a) adipogenesis↓; (b) lipolysis↑;	C/EBP-β And SREBP1c↓ ATGL↑	32
C57BL / 6 mice	200mg / kg	(a) blood glucose↓; (b) insulin resistance↓ ; (c) Regulating intestinal flora	intestinal flora ↑	33
SHEPG2 cells (liver) , THP-1 cells (marcophages, Caco2 cells (intestine)	10μM,20μM	(a) hepatic triglyceride accumulation↓;	Akt and SREBP-1↓ Akt-MTORC1dependent autophagy pathway.	34
THP-1 cells.	2.5μg / ml; 5 μg / ml; 10 μg / ml;	(a) Macrophages lipid accumulation ↓ ; (b) Prevention of atherosclerosis;	CD 36↓ ABCA1, ABCG1↑	35
*In vitro*: HeLa cells, 3T3-L1cells. *In vivo*: Rats.	20μM; 10 mg/kg	(a) Lipid autophagy↑; (b) lipid droplet degradation↑; (c) lysosomal Ca2 + efflux ↑; (d) TFEB translocation↑;	ATG5-ATG12 ↑ TFEB ↑ TUFM ↑	36
3T3-L1 cells	60μM	(a) lipolysis↑; (b) lipid accumulation↓; (c) fat differentiation ;	CEBP- α↓ PNPLA2 and LIPE ↑	28
Male TSOD mice and TSNO mice	5mg / kg 15mg / kg	(a) lipid synthesis↓; (b) fatty acid oxidation↑; (c) liver cholesterol transport↑;	LXR, SREBP - 1C↓ PPAR α↑ ApoA1 ↑	37
HepG2 cells	5μM, 10μM, 20μM	(a) lipid accumulation ↓; (b) oxidative stress ↓;	SREBP1, FASSCD-1↓ PPARγ , C/EBP-β↓ HO-1/Nrf2↓	38
3T3-L1 cells	50μmol/L	(a) the early stage of adipogenesis↓;	MCE↓ apoptosis↑	34
MaleC57BL/ 6 J mice	10mg/kg	(a) lipid metabolism↑ ; (b) glucose metabolism↑;	PPARγ/LXRα/ABCA1↑ PPARγ/PI3K/AKT↑	39
*In vivo*: db/db mice, *In vitro*: MIN6 pancreas β Cells	50μM, 100μM	(a) lipid metabolism↑ ; (b) glucose metabolism↑; (c) β-cell proliferation↑;	SREBP-1↓ IRS/PI3K/AKT↑ IRS2/FOXO1↑	40
ApoE**^-^** C57BL / 6J male mice	150 mg / kg	(a) plasma glucose↓ (b) insulin sensitivity↑; (c) high-density lipoprotein cholesterol levels↑;	LXR-β↑ Akt , SREBP-1↓	41
Male Wistar rats.	50, 100, and 200 mg/kg	(a) blood glucose↓; (b) antioxidant↑;	lipid peroxidation↓	42
Male Wistar rats.	100mg/kg	(a) membrane-bound ATPases↑; (b) antioxidant↑;	–	43, 44
Yeast glucosidase	Kaempferol solution (6.82 * 10-6mol / L)	(a) Blood glucose↓;	α-glucosidase↓	45
Male C57BL/6J mice	50mg/kg	(a) hepatic gluconeogenesis↓; (b) glycogen synthesis↑; (c) Blood glucose↓;	PC and G6P ↓ Akt and GCK↑	27, 46
RIN-5F cells	1μM, 10μM and 50μM	(a) lipotoxicity l↓; (b) pancreatic β-cells apoptosis↓;	AMPK/mTOR↑	47
IN-5F cells	10μM	(a) Lipophagy↑; (b) ER stress↓; (c) β-cell mass and function↑;	AMPK/mTOR↑	48
HeLa cells	1μM	(a) Anti diabetes and diabetic complications;	Ca2+ uniporter↑	49
INS-1E β Cells,	0.1μM, 1μM and 10uM	(a) pancreatic β-cells apoptosis↓; (b) β-cells secrete insulin↑;	PDX-1/cAMP/PKA/CREB↑ MCU↑	21, 50
INS-1E β Cells, Human islet (CMRL-1066) cells	0.01μM, 0.1μM, 1 μM and 10uM	(a) apoptosis↓; (b) pancreatic β-cells↑;	caspase-3↓ Akt and Bcl-2 ↑	51

“↑” refers to upregulation, and "↓" refers to downregulation.

## Anti-lipotoxicity effect of kaempferol

With the lipid accumulation in adipose tissues during obesity, fat macrophages release inflammatory cytokines, such as TNF-α and IL-6. In insulin-sensitive organs, these cytokines stimulate c-Jun amino terminal kinase (JNK) and IκB kinase-β/nuclear factor-κB (NF-κB) pathways, blocking insulin signaling. IR results in T2DM (52). In IR, the function of insulin in inhibiting lipolysis is impaired, and free fatty acids (FFA) levels increase. Subsequently, these fatty acids are deposited in insulin-sensitive organs and tissues, such as the liver, skeletal muscle, and pancreas. Lipid metabolism disorders are a charecteristics of T2DM.

According to a previous study, an increase in circulating FFA levels occurred earlier than glucose intolerance (53). FFAs are transported to the liver and metabolized into acetyl coenzyme A (CoA), which enhances the activity of pyruvate carboxylase (PC) and provides a substrate for gluconeogenesis (54). Glycerol released from lipolysis is also a direct substrate for hepatic gluconeogenesis (55). One of the mechanisms by which metformin inhibits diabetes is the inhibition of gluconeogenesis using glycerol as a substrate. The “glucose fatty acid cycle” is usually used to describe glucose metabolic damage induced by FFAs (56). Theoretically, hyperglycemia can be corrected by removing the excessive accumulation of ectopic lipids (57, 58).

### Anti-adipogenic effect of kaempferol

Chronic overnutrition and obesity are usually associated with IR and hepatic steatosis (fatty liver) (59). Under nutrient-rich conditions, the expression and transcription of fatty acid synthesis genes are upregulated, leadig to increased fatty acid synthesis. Sterol regulatory element-binding proteins (SREBPs) are adipogenic transcription factors that contain two subtypes SREBP1 and SREBP2. SREBP1c is the main subtype expressed in most tissues, whereas SREBP1a is highly expressed only in some tissues and cells (heart, macrophages) (60). SREBP2 regulates cholesterol metabolism (61). SREBP2 activity is controlled by downstream products of the cholesterol biosynthesis pathway with a highly regulated negative feedback mechanism (62). When SREBP2 is transferred to the nucleus, it activates the expression of cholesterol-related genes, such as 3-hydroxy-3-methylglutaryl-CoA reductaseand low-density lipoprotein receptor (LDLR) (63). Nevertheless, the mechanism by which SREBP2 regulates cholesterol homeostasis is complex, and there may be different regulatory mechanisms in different organs. In the liver, berberine inhibits hepatic cholesterol deposition by downregulating the silent mating type information regulation 2 homolog (SIRT1)-forkhead box protein O1 (FOXO1)-sSREBP2 pathway (64). In the aorta, metformin inhibits SREBP2-LDLR-mediated aortic cholesterol uptake by activating activated protein kinase (AMPK) (65). However, there are no direct experimental data to confirm that SREBP2 is a mechanism by which kaempferol regulates lipid metabolism disorders. Notably, this aspect deserves further study.

**Figure 2:**
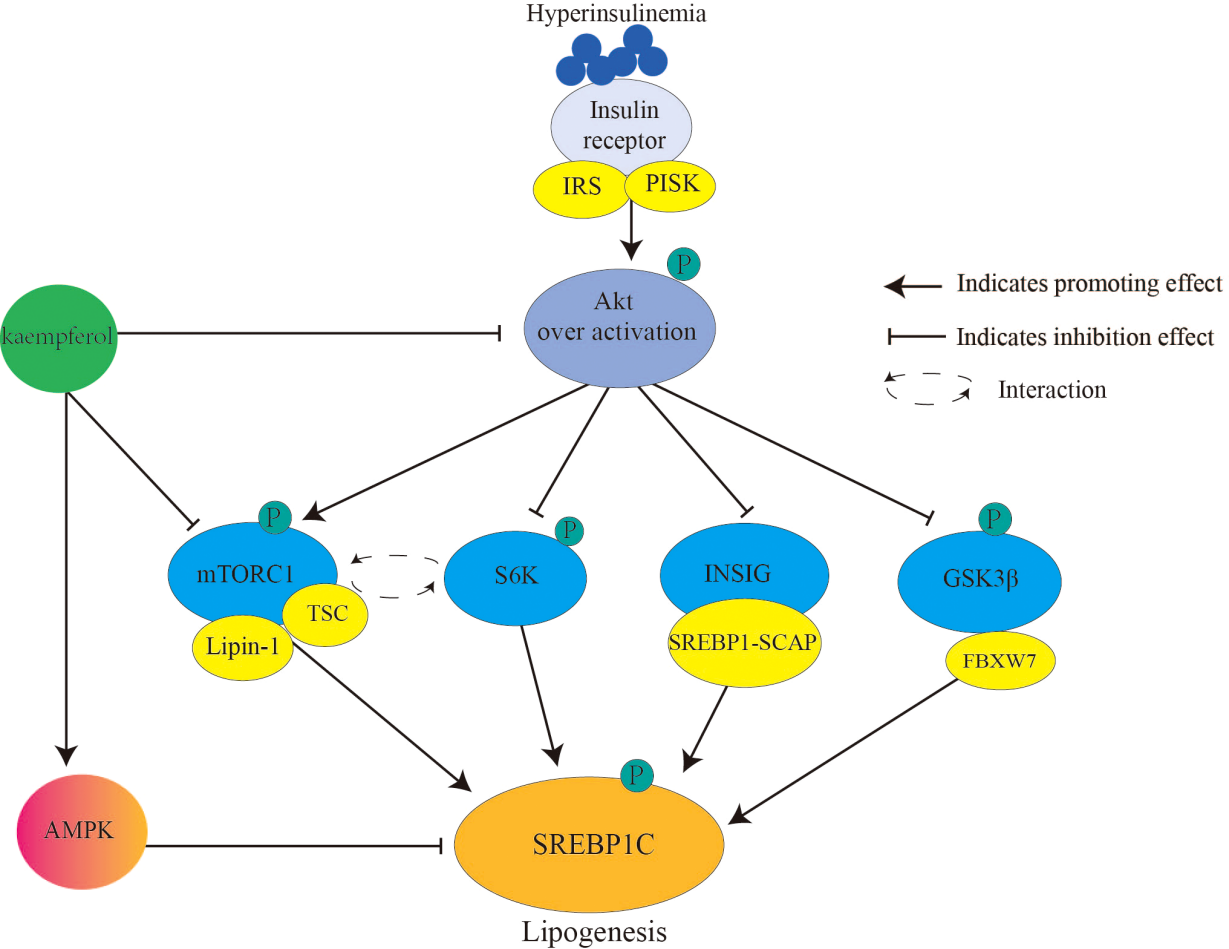
Kaempferol reduces SREBPlc to inhibits lipogenesis. Hyperinsulinemia over activates Akt the downstream signaling targets of insulin. Therefore, it causes the activation of AKT/mTORC1/SREBP1C signal and lipogenesis. Kaempferol inhibits the activation of Akt and mTORC1, thereby blocking the activation of the downstream signal SREBP1C. In addition, kaempferol directly activates AMPK to inhibit SREBP1C mediated adipogenesis. IRS, Insulin receptor substrate; PI3K, inosine phosphate 3-kinase; AKT; threonine protein kinase; mTORCl: rapamycin complex 1; S6K, ribosomal protein S6 kinase; INSIG: insulin induced target gene protein; GSK3β, Glycogen synthesis kinase 3β; TSC, tuberous sclerosis; SREBP1, sterol regulatory element binding proteinsl; SCAP, SREBP cleavage-activating protein; FBXW7, F-box and WD repeat domain containing 7; AMPK, AMP-activated protein kinase.

SREBP1c (**Figure 2**) primarily controls the expression of adipogenic genes and regulates fatty acids. During IR, chronic hyperinsulinemia overactivates the liver protein kinase B (Akt)/mammalian target of rapamycin complex 1 (mTORC1)/SREBP1c pathway, inducing excess adipogenesis (66). This is one of the causes of lipid metabolism disorders in patients with diabetes. In detail, Akt (threonine protein kinase) phosphorylates and inhibits insulin-induced gene (INSIG2), which transports SREBP-SREBP cleavage-activating protein complex to the Golgi apparatus for proteolytic activation (67). Akt phosphorylates and inhibits glycogen synthase kinase 3 (GSK3) β/F-box and WD repeat domain containing 7-mediated ubiquitin precursor system, thereby reducing SREBP degradation (68). mTORC1 promotes SREBP1 activation and lipid synthesis by interacting with ribosomal protein S6 kinase (S6K) (69). Moreover, mTORC1 inhibits tuberous sclerosis complex (TSC) (an upstream inhibitor of mTORC1) through Akt-mediated phosphorylation, whereas phosphorylated mTORC1 secretes the nuclear phosphatase lipin-1, thereby activating nuclear SREBP1c (70). Once SREBP1c is activated, insulin signaling is blocked (71).

Kaempferol downregulates SREBPs and upregulates liver peroxisome proliferator-activated receptor α (PPARα), promoting the expression of propyl CoA oxidase and cytochrome P450 isomer 4A1 reducing the accumulation of visceral fat, and improving hyperlipidemia in high-fat diet-fed obese rats (31). Kaempferol upregulates liver X receptor (LXR), which regulates lipid transport (37, 41). Therefore, kaempferol exhibits a strong lipid-regulating effect in different cell types. In adipocytes, differentiated from human mesenchymal stem cells, kaempferol downregulates the CCAAT enhancer binding protein (C/EBP) β and SREBP1c, and upregulates the expression of adipose triglyceride (TAG) lipase (ATGL) to inhibit the accumulation of TAGs (32). In 3T3-L1 cells, kaempferol inhibits TAG synthase (such as LPAATθ, lipon-1[LPIN1] and diacylglycerol [DAG] acyltransferase 1), fatty acid synthase (FASN), and SREBP1c related fatty acid synthesis to inhibit lipid accumulation (30). Kaempferol inhibits mitotic clonal expansion and induces apoptosis in the early stages of adipogenesis (34). In HepG2 cells, kaempferol inhibits Aktactivity and SREBP1 through a variety of mechanisms, increasing the expression of INSIG-2a, reducing SREBP1 phosphorylation, and increasing GSK-3 phosphorylation (72).

### Kaempferol regulates lipolysis and transport

Under the condition of excess energy, fuels are stored in adipocytes as TAGs. The fat storage capacity of adipocytes prevents lipotoxic damage (lipid-induced dysfunction and programmed cell death) in tissues and organs (especially the skeletal muscle, liver, and pancreas) (73). Lipolysis is defined as the decomposition of TAGs in lipid droplets into glycerol and non-acylated fatty acids (NEFAs) with the release of energy (74). The main hormones that regulate lipolysis are catecholamines and insulin. Catecholamines promotes lipolysis, whereas insulin inhibits lipolysis. Insulin regulates the uptake of glucose and fatty acids in adipocytes and triggers the translocation of fatty acid transport (75). Insulin strongly inhibits basal lipolysis and catecholamine-induced lipolysis by activating phosphodiesterase-3b (PDE-3B) through PKB/Akt-dependent phosphorylation (76). PDE-3B reduces cyclic adenosine monophosphate (cAMP) levels, downregulates protein kinase A (PKA) activation, and reduces PKA-stimulated hormone-sensitive lipase (HSL) phosphorylation by catalyzing cAMP decomposition into inactive forms (76). Insulin also activates the regulatory subunit of protein phosphatase-1 by phosphorylation, causing rapid HSL dephosphorylation and inactivation (77).

In addition, inflammatory cytokines such as TNF-α, IL-6, and IL-1β, secreted by adipocytes and adipocytes promote lipolysis. The mechanisms by which TNF-α promotes lipolysis are as follows. First, insulin signaling is inhibited by tyrosine phosphorylation of insulin receptor substrate 1. Perilipin is a protective protein around lipid droplets that prevents lipid droplets from being decomposed by HSL (78). However, TNF receptor 1 reduces perilipin through mitogen-activated protein kinase (MAPK) (p44/42, JNK) (79). IL-1β is another significant pro-inflammatory cytokine that is mainly produced by macrophages. In human adipocytes, IL-1β inhibits insulin signal transduction and glucose transporter type 4 (GLUT4) at doses as low as 2 ng/ml (80). During IR, the inhibition of perilipin allows lipase to enter lipid droplets. After lipase activation, TAGs are decomposed in three steps. Initially, TAGs are hydrolyzed by ATGL to produce fatty acids and DAG (81). HSL catalyzes the hydrolysis of DAG to monoacylglycerol (MAG) and fatty acids (82). Finally, MAG lipase hydrolyzes MAG to fatty acids and glycerol (83).

It differs from hyperlipidemia caused by excessive lipolysis during inflammation and IR. The ultimate goal of kaempferol in the regulation of lipid metabolism is to reduce ectopic lipid deposition and maintain lipid homeostasis. Kaempferol at 60 μM stimulates 62% inhibition of adipogenesis in preadipocytes and results in a 39% reduction in intracellular lipid accumulation in mature adipocytes (28). The PPARβ/δ signaling cascade regulates the expression of PPARγ and C/EBP family (84, 85). PPARγ and C/EBP are pivotal transcriptional regulators of lipid homeostasis, regulating the expression of FASN, ATGL, and HSL (29, 86). Kaempferol downregulates PPARγ and C/EBP-β to activate ATGL and directly promotes lipolysis in a concentration-dependent manner (29). Patatin-like phospholipase domain containing 2 (Pnpla 2) and Lipe also encode ATGL and HSL, respectively (87). In 3T3-L1 cells, kaempferol upregulates the mRNA expression of Pnpla 2 and Lipe, indirectly increasing the expressions of ATGL and HSL (28). LPIN1, a co-regulator of DNA-bound transcription factors, is highly expressed in adipocytes and functions as a phosphatidic acid (PA) phosphatase enzyme that dephosphorylates PA to DAG (88). LPIN1, PPARγ coactivator α, and PPARα synergistically regulate fatty acid oxidation gene expression (89). Kaempferol downregulates the protein level of LPIN1 in a dose-dependent manner (29), which seems to be related to the downregulation of PPARγ. LXR maintains cholesterol homeostasis by regulating cholesterol efflux, transportation, and absorption (90). The use of LXR agonists (fibrates) activates SREBP1c, eventually leading to fatty liver and hypertriglyceridemia (59). Kaempferol activates LXR, especially the β subtype, which lowers cholesterol and glucose levels in apolipoprotein E-deficient mice (41). However, the expression of LXR β is not highly in the liver; therefore, the selective activation of LXR β by kaempferol does not cause an increase in SREBP1c. In macrophages, lipids are absorbed by scavenger receptor A and cluster of differentiation 36 (CD36) (91). Cholesterol is transported to the outside of the macrophages by reverse cholesterol transporters, including scavenger class B type I (SR-BI), ATP-binding cassette transporter A1 (ABCA1), and ATP binding cassette transporter G1 (ABCG1) (92, 93). Kaempferol downregulates CD36 and upregulates SR-BI, ABCA1, and ABCG1 (35). The phagocytosis of lipid-forming foam cells by macrophages is an early marker of atherosclerosis. Therefore, kaempferol is likely to have anti-atherosclerotic effects. Similar to other phenolic substances different kinds of kaempferol have strong anti-inflammatory effects. Kaempferol glycosides inhibit the expression of the transcription factor PPARγ and decrease TNF-α levels (94). In contrast, kaempferol inhibits the pro-inflammatory signals of TNF-α, IL-6, IL-1β, and NF-κB (95–97). Evidently, anti-inflammation is also a good measure to regulate metabolic disorders and diabetes.

## Kaempferol improves glucose metabolism

### Insulin resistance and hyperglycemia

The discovery and administration of insulin are milestones in the treatment of diabetes, which transforms diabetes from a life-threatening disease to a controllable disease (98). Insulin binds to the insulin receptor on the outer surface of the cell, causing tyrosine phosphorylation of insulin receptor substrate, and then binds to inosine phosphate 3-kinase to form phosphatidylinositol (3,4,5)-triphosphate (PIP3). PIP3 activates Akt, 3-phosphoimide dependent protein kinase 1, which then activates P70 ribosomal S6 kinase (S6K) and protein kinase C (99). Akt-dependent phosphorylation plays an important role in the physiological effects of insulin. First, Akt-induced phosphorylation causes GSK3α/β inactivation, which leads to dephosphorylation and activation of glycogen synthase (100). Second, Akt phosphorylates theTBC1 domain family member 1/AKT substrate of 160 kDa (TBC1D4/AS160) to regulate the transport of intracellular GLUT4 vesicles to the cell membrane to increase glucose uptake (101, 102). Third, Akt phosphorylation of TSC2 leads to mTORC1 activation, which stimulates lipid and protein synthesis and inhibits autophagy (103, 104). Fourth, Akt phosphorylates and inhibits the translocation of FOXO transcription to the nucleus, thereby inhibiting liver glucose-producing gene and muscle autophagy gene expressions and lipolysis (105, 106). Interestingly, in contrast to insulin inhibition of FOXO transcription, insulin induces Akt and mTORC1 activation by inhibiting GSK3α/β, thus inhibiting forkhead box class K (FOXK) phosphorylation and causing FOXK nuclear localization and transcription (107). FOXK regulates the expression of genes involved in cell cycle, apoptosis, and lipid metabolism and even stimulates glycolysis (108).

Insulin plays a role in regulating metabolism in different insulin sensitive-tissues through subsequent recognition signal transduction with insulin receptor substrates and tyrosine kinase activity (109). In the skeletal muscles, insulin promotes glucose transport and utilization, stimulates glycogen synthesis, and inhibits protein decomposition. In adipose tissues, insulin promotes glucose transport and lipogenesis, and inhibits lipolysis. In the liver, insulin inhibits gluconeogenesis and fatty acid oxidation and stimulates glycogen synthesis and lipogenesis (*de novo* lipogenesis) (110). In IR, hepatic gluconeogenesis and hepatic glucose production are increased. However, glycogen synthesis and glucose uptake are blocked, resulting in an increased hepatic glucose output and elevated blood glucose levels.

### Multi-target hypoglycemic effects of kaempferol

**Figure 3:**
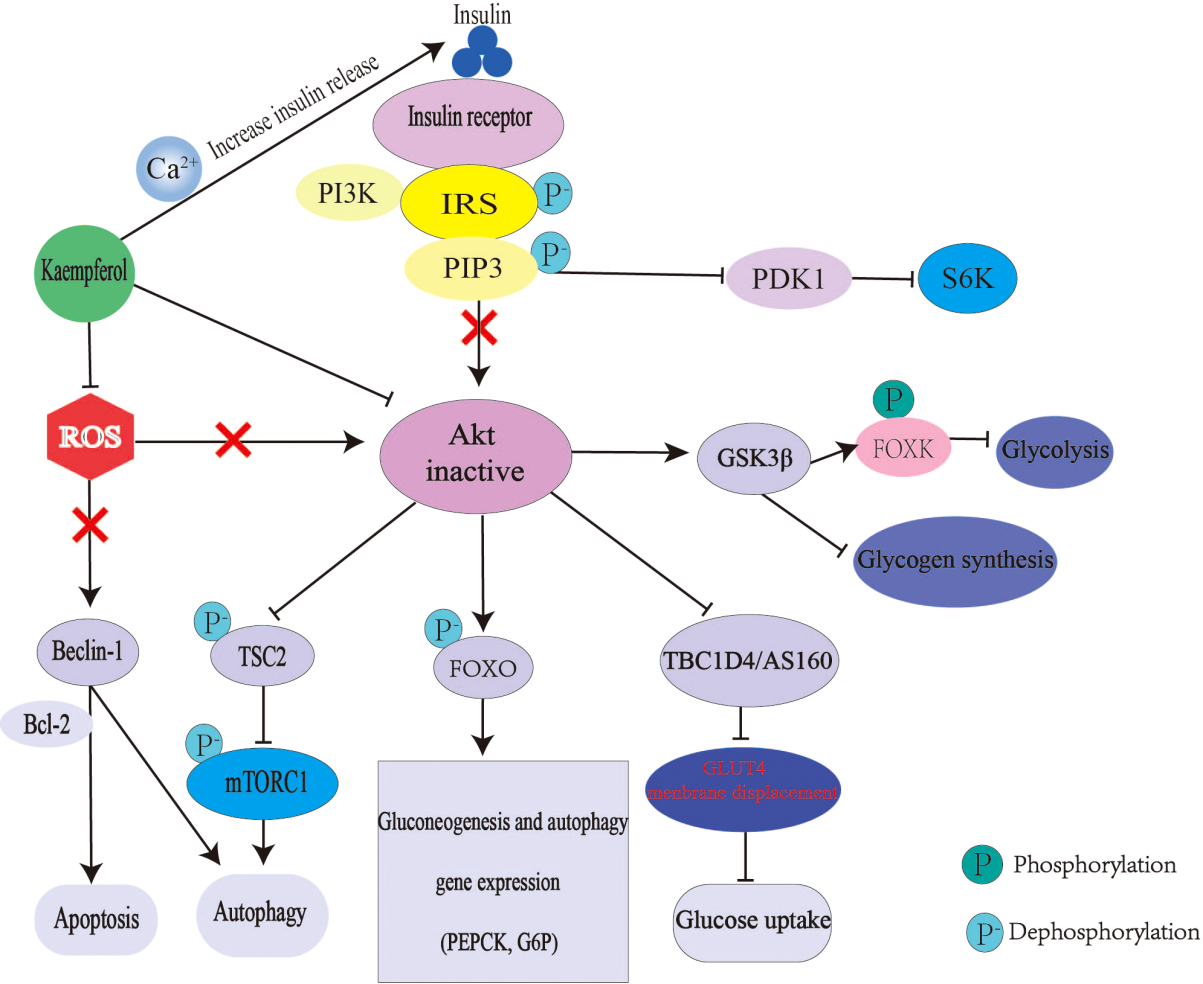
Mechanism of kaempferol hypoglycemic. In diabetes, insulin signal transduction is blocked. The expression of gluconeogenesis gene was up-regulated and liver glucose output was excessive. The decrease of glycogen synthesis and glucose uptake makes glucose output greater than consumption, which leads to hyperglycemia. Kaempferol promote insulin secretion and improve Akt activity by regulating mitochondrial calcium uptake. Kaempferol can also directly restore the activity of Akt. Thus reversing the up regulation of gluconeogenesis, down regulation of glycogen synthesis and glucose uptake caused by Akt inactivation. Moreover, kaempferol antioxidant can also regulate autophagy and apoptosis. IRS, Insulin receptor substrate; PI3K, inosine phosphate 3-kinase; AKT; threonine protein kinase; PIP3, phosphatidylinositol 3,4,5-trisphosphate; PDK1, 3-phosphoinositide-dependent protein kinase 1; S6K,ribosomal protein S6 kinase; GSK3 β, Glycogen synthesis kinase 3 β ; FOXO, Forkhead box 0; FOXK, Forkhead Box Class K; TSC2, tuberous sclerosis 2; ROS, reactive oxygen species; Tbc1d4/AS160, Akt substrate of 160 kDa; BCL-2, B cellleukemia/lyrnphoma-2; mTORC1, rapamycin complex1; PEPCK, phosphoenolpyruvate carboxylase; G6P, glucose-6-phosphatase.

Relative or absolute deficiency of insulin secretion and insulin action is the basic pathogenesis of diabetes. Kaempferol can interfere with the above process through many ways (**Figure 3**). Kaempferol promotes insulin secretion, which is similar to insulin secretagogue. A study using glibenclamide, an insulin secretagogue, as the control drug, found that kaempferol increased the plasma insulin levels and reduced blood glucose levels in streptozotocin-induced diabetic rats (43). Mitochondrial Ca^2+^ plays an important role in insulin release and glucose metabolism (111). Mitochondrial calcium monoporter (MCU) is the main pathway of Ca^2+^ uptake in the mitochondria (112). Kaempferol directly activates MCU in a concentration-dependent manner. only 1μM can nearly double the uptake of mitochondrial Ca^2+^ and then activate the pancreatic β-cell metabolism/secretion coupling (49, 50). In a C57BL/6 mouse model of diabetic nephropathy (DN), kaempferol increased glucagon-like peptide 1 (GLP-1) and insulin levels with an increase in cAMP, Ca^2+^ and glutathione (GSH) levels (113). Kaempferol also improves insulin-dependent glucose uptake in 3T3-L1 adipocytes and pig myotubes (114, 115).

An imbalance in glucose production and utilization causes glucose metabolism disorders. Hepatic IR is an important cause of fasting hyperglycemia. Under the condition of hepatic IR, glucose metabolism-regulating enzymes levels, such as glucose-6-phosphatase, PC, glucokinase (GCK) and phosphoenolpyruvate carboxykinase (PEPCK), are abnormal. The activation and inactivation of GCK are closely related to blood glucose levels. Therefore, GCK activator is a potential target for the treatment of diabetes (116). Kaempferol reduces blood glucose levels by increasing GCK levels and promoting glycogen synthesis (46). In mice, oral administration of kaempferol (50mg/kg/day) significantly improves hyperglycemia by restoring the activity of hexokinase, while inhibiting the activity of PC and gluconeogenesis, thus reducing the morbidity rate of diabetes from 100% to 77.8% (27). When insulin signaling is activated, Akt phosphorylates and inhibits FOXO1 transcription, ultimately inhibiting PEPCK and G6P expressions (117, 118). The mechanism by which kaempferol inhibits hepatic gluconeogenes is also includes a direct increase in Akt activity and PC inhibition (46). Kaempferol inhibits the hepatic inhibitor IκB kinase/NF-κB pathway as its anti-inflammatory effect and restores Akt activity (119).

Adenosine 5’-monophosphate (AMP)-AMPK is one of an important energy sensor and the main regulator that maintains systemic metabolic homeostasis (120). IR is accompanied by a sustained decrease in AMPK activity, which increases insulin sensitivity (121, 122). AMPK inactivates acetyl CoA carboxylase (ACC) by phosphorylation, thus preventing malonyl-CoA synthesis, increasing mitochondrial fatty acid oxidation, and reducing fatty acids synthesis (123). AMPK activation is an important pharmacological target for diabetes treatment. Metformin and thiazolidinediones (TZDs) have been identified as AMPK activators (124, 125). Kaempferol increases the phosphorylation of AMPK and ACC in the adipose tissues, liver, and muscles (126, 127). Therefore, kaempferol is possibly the same as metformin and TZDs as a direct activator of AMPK.

α-Glucosidase hydrolyzes glucoside bonds to glucose, which plays an important role in carbohydrate metabolism, and is therefore an attractive therapeutic target for the treatment of diabetes, obesity, and metabolic syndrome (128). Kaempferol is a novel α-glucosidase inhibitor. Kaempferol blocks its catalysis to glucoside by inserting into the active site of α-glucosidase, occupying the catalytic center of the enzyme and inducing conformational changes (45). Therefore, foods rich in kaempferol can reduce carbohydrate absorption and reduce postprandial glucose levels. Change in the intestinal microbiota are important in the pathogenesis basis of obesity, type 2 diabetes, and metabolic syndrome (129).

Kaempferol reduces the relative abundance of thick-walled flora, increases the level of *B*acteroides, reduces blood lipids and glucose levels, and improves IR in obese C57BL/6 mice (33).

## Kaempferol protects pancreatic β cells

Most patients with diabetes experience β cells mass loss and apoptosis (17). Pro-inflammatory factors, such as IL-1β, interferon and TNF-α induce β-cell apoptosis. The caspase-dependent intrinsic apoptotic pathway is the innitial effector of inflammatory β cells apoptosis (130). TNF-α and IL-1β induce NF-κB activation (131). NF-κB then reactivates inducible nitric oxide synthase expression, which subsequently causes the release of nitric oxide (NO) (132). NO-dependent Ca^2+^ depletion in the endoplasmic reticulum (ER) leads to ER stress, C/EBP homologous protein (CHOP) induction, and finally β Cells apoptosis (133, 134). However, the proapoptotic and antiapoptotic effects of NF-κB activation are controversial among different cell types. The activation of NF-κB promotes apoptosis after exposure to IL-1β or TNF-α in β cells (131). The IL-1β/NF-κβ pathway is considered the “common pathways” of β cells death in types 1 and 2 diabetes (135).

Autophagy is defined as an intracellular lysosomal degradation process of defective proteins, macromolecules, damaged organelles, and toxic aggregates and plays a crucial role in maintaining intracellular balance (136). Autophagy disorders are associated with IR, obesity, and T2DM (137, 138). Exposure of human islets β Cells to fatty acids and high glucose levels leads to apoptotic cell death by preventing autophagic flux (139). Overactivation of autophagy is related to an increase in lipolysis, resulting in ectopic lipid deposition and lipotoxic damage in β cells.

**Figure 4:**
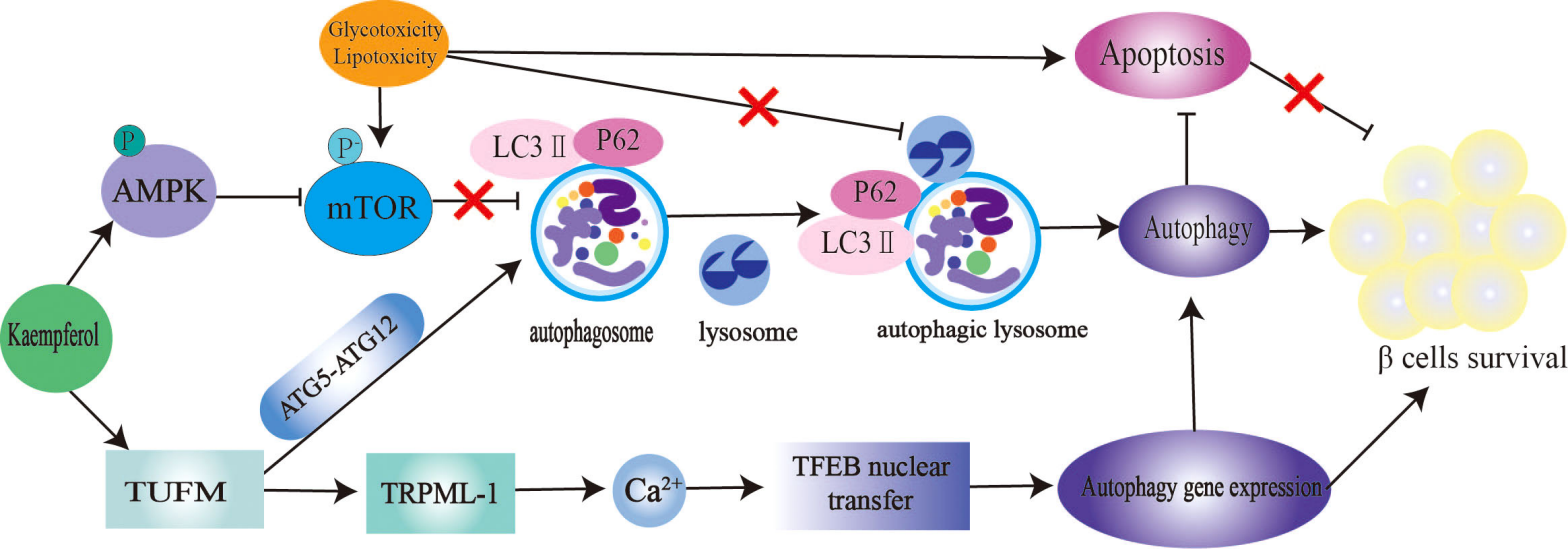
Kaempferol stimulates autophagy to protect pancretic β Cells. Kaempferol up regulates intracellular lipid autophag of β cells by activating AMPK/mTOR signal pathway and TUFMTFEB signal pathway. Thus inhibiting β Apoptosis, restore autophagy-apoptosis balance, and protect pancreas β Cells. AMPK, AMP-activated protein kinase; mTOR, rapamycin; LC3, microtubule-associated protein light chain 3; ATG5, autophagy-related geneS; ATG12, autophagy-related gene 12; TUFM, Tu translation elongating factor, mitochondrial; TRPML-1, transient receptor potential mucolipin 1; TFEB, nuclear translocation of transcription factor.

The mTOR/AMPK pathway and autophagy-related genes (ATGs) play a significant role in the regulation of autophagy (140). Microtubule-associated protein light chain 3-II (LC3-II) and p62 are markers of autophagy. Inhibition of the autophagy negative regulator mTOR improves IR and hepatic steatosis in T2DM rats (141). Kaempferol is an excellent autophagy enhancer (**Figure 4**). The activation of autophagy induced by kaempferol promotes intracellular lipid degradation, reduces ER stress,and protects β cells from lipotoxic damage (142). Kaempferol interacts with Tu translation elongating factor, mitochondrial (TUFM). TUFM enhances the interaction between ATG12–ATG5 complexes, thereby promoting the formation of autophagosomes and lysosomes. Transient receptor potential mucolipin 1 (TRPML-1) is a permeable cation selective channel that promotes intracellular calcium release (143). Mitochondrial reactive oxygen species (mtROS) regulates TRPML-1-mediated lysosomal Ca^2+^ release (143). As an autophagy enhancer, kaempferol induces mtROS to promote lysosomal Ca^2+^ efflux, transcription factor EB translocation, and autophagy induction (36). In this study, the authors believe that the enhanced autophagy of 3T3-L1 and HeLa cells induced by kaempferol is not related to AMPK-mTOR signaling (36). In contrast, in RIN-5F cells treated with palmitic acid, 10 μM kaempferol increased AMPK phosphorylation, decreased mTOR phosphorylation, reduced caspase-3 cleavage by approximately 2.5 times, and reduced the mortality rate of RIN-5F cells from 32% to 2% (47). In another study, 10 μM kaempferol treatment increased the colocalization of lipid droplets with autophagosomes and lysosomes in cells through AMPK-mTOR signaling and reduced ectopic lipid accumulation and ER stress (48). Kaempferol also plays an anticancer role by activating the IRE1/JNK/CHOP signaling pathway (144).

Diabetes is also closely associated with oxidative stress. Since oxidative stress was observed in experimental diabetes in the 1980s (145), the role of oxidative stress in the pathogenesis of diabetes and its complications has attracted extensive discussion in academia. In diabetes, persistent hyperglycemia eventually leads to excess production of reactive oxygen species (ROS) by increasing mitochondrial oxygen consumption, destroying mitochondrial function, or activating nicotinamide adenine dinucleotide phosphate oxidase (146). Excess ROS-induced β Cells dysfunction and IR are the main causes of diabetes and its complications. Consistent with other natural products, kaempferol has an excellent antioxidant effect. In diabetes, kaempferol prevents pancreatic β cells oxidative damage (40, 42, 51, 147). This may be related to kaempferol restoring the levels of nonenzymatic antioxidants (vitamins C and E, reduced GSH) and enzymatic (superoxide dismutase, catalase, GSH peroxidase, and GSH-S-transferase) antioxidants to reduce glucose and lipid peroxidation in β cells (43). Kaempferol prevents myocardial hypertrophy by inhibiting the ASK1/MAPK signaling pathway, regulating oxidative stress, improving cardiac function, and reducing apoptosis (148). In male albino Wistar rats, the use of kaempferol reversed the increase in γ-glutamyl transferase and lipid peroxidation marker (thiobarbituric acid reactive substances and lipid hydroperoxides) levels (149). In rats with cerebral ischemia/reperfusion injury, 10–15 μmol/L kaempferol reduces nitrous oxidative stress after ischemia/reperfusion and inhibits apoptotic cell death and apoptotic biochemical markers (such as caspase-9 activity and poly [ADP-ribose] polymerase [PARP] degradation) for brain protection (150).

Diabetes-related hyperlipidemia and changes in membrane phospholipids and fatty acids inhibit membrane-bound enzyme activity (151). Membrane-bound ATPase contains Na^+^/K^+^-ATPase, Ca^2+^-ATPase, and Mg^2+^-ATPase, which are channels for cations to enter and leave cells. It plays an important role in maintaining cell physiological functions and is closely related to pathological changes. The use of kaempferol in diabetic rats significantly increases the activity of membrane-bound ATPases in the erythrocyte, liver, kidney, and heart tissues (44). This is another mechanism by which kaempferol protects β cells.

## Kaempferol’s role in antidiabetic complications

### Diabetic retinopathy

Diabetes is usually associated with one or more complications. Diabetic retinopathy is a common microvascular complication. In hyperglycemia-induced retinal ganglion cell (RGC) injury, 60 μmol/L of kaempferol reduces RGC cell damage and improves cell survival by increasing extracellular signal-regulated kinase phosphorylation and vascular inhibitor protein 1 expression (152). Retinal pigment epithelium (RPE) damage is associated with diabetic retinopathy progression. Oxidative stress caused by glucotoxicity and lipotoxicity is the main inducer of RPE injury. Kaempferol inhibits vascular endothelial growth factor mRNA expression and the bax/bcl-2/caspase-3 signaling pathways, thus protecting human RPE cells (ARPE-19) from hydrogen peroxide-induced injury and apoptosis (153). A prior large-scale study found that kaempferol at a dosage of 5–25 μm has anti-angiogenesis effects (154). Interestingly, kaempferol inhibits estrogen-related receptors α, thus also inhibiting the angiogenesis of human retinal endothelial cells (155).

### Diabetic nephropathy

DN is the most prevalent diabetes complications with the highest prevalence and accounts for 30–47% of all kidney diseases (156). Damage to glomerular mesangial cells (GMCs) is a key risk factor for early-stage DN. The interaction between advanced glycation end products (AGEs) and their receptors (RAGE) is an important mechanism of GMC damage. Kaempferol protects GMC to prevent DN. The mechanisms of kaempferol in DN include inducing antioxidative stress, inhibiting collagen IV and transforming growth factor- β1, improving mitochondrial membrane potential, and inhibiting the mitochondrial/cytochrome C-mediated apoptosis pathway (157). In hyperglycemia-induced podocyte apoptosis, kaempferol regulates M1/M2 polarization of glomerular macrophages and reduces TNF-α and IL-1β levels (3). Glomerular matrix fibrosis is another predisposing factor for DN progression. Kaempferol promotes the release of GLP-1 and insulin while inhibiting RhoA/Rho kinase and fibrosis (113). Chronic inflammation is also a key factor in DN progression. Kaempferol blocks Toll-like receptor 4, and NF-κB by downregulating TNF receptor-associated factor 6 to reduce the inflammatory response in DN (158).

### Diabetic cardiomyopathy

Diabetic cardiomyopathy (DCM) is a major cardiovascular complication of diabetes that leads to heart failure and even death (159). Increased production of ROS, inflammation, cardiomyocyte apoptosis, and myocardial fibrosis are involved in the pathogenesis of DCM (160). Kaempferol upregulates SIRT1 (161), inhibits NF-κB nuclear translocation, and activates nuclear factor E2-related factor (162), thereby inhibiting diabetes-induced myocardial inflammation and oxidative stress. Kaempferol inhibits ASK1/MAPK signaling and regulates oxidative stress to prevent cardiac hypertrophy (148). In diabetes myocardial ischemia/reperfusion injury, kaempferol reduces the oxidative stress and inflammation induced by AGE-RAGE/MAPK to alleviate myocardial ischemia/reperfusion injury (163).

Moreover, kaempferol promotes wound healing in patients with and without diabetes by promoting wound reepithelialization (164). In DN, kaempferol partially reverses pain sensitivity by regulating oxidative and nitrosative stresses and reducing AGEs formation (165).

## Conclusion and outlook

Obesity and diabetes are two chronic inflammatory diseases. NF-κB activation plays an important role in these diseases. This is inseparable from PARP1 (166, 167). PARP activation induced by lipotoxicity in the liver causes a decrease in NAD^+^, SIRT1, LXR, and AMPK levels and insulin receptor activation (168, 169). Inhibiting PARP1 prevents β-cell death (170, 171). Kaempferol activates AMPK and PPARα; suppresses C/EBP-α, SREBP1c, and PPARγ; and protects β Cells (38, 39). Therefore, kaempferol may serve as a natural PARP inhibitor.

Low plasma concentrations of kaempferol restrict its use. In fact, the flavonoid naturally absorbed by the human body is only 1–2 g per day, and the plasma concentration is in the range of micromolars (172). However, kaempferol at concentrations as low as 1 μM increases mitochondrial Ca^2+^ uptake by approximately 85% (49). A previous *in vitro*, study found that kaempferol was not cytotoxic at a concentration of 60 or 75 μM (28, 47). Low bioavailability is another limiting factors for clinical studies on kaempferol (173, 174). The body has a defense mechanism that excludes foreign objects through cell membrane surface receptors (173). Interestingly, two methods that may improve the bioavailability of kaempferol. The first method is binding to another substance with higher affinity for the transporter protein, which transports substances with higher affinity to the outside of the cell, while those with lower affinity remain and continue to work. This method was validated for the binding of kaempferol and quercetin to the breast cancer drug resistance protein (ABCG2). The affinity of kaempferol for ABCG2 was higher than that of quercetin. Therefore, ABCG2 transports kaempferol to the extracellular spaceand leaves quercetin *in vivo* (175). The second method is to use nanocarriers to increase permeability and achieve systemic circulation by coating nanoparticles on the surface of kaempferol. Nanoparticle capsules may to protect kaempferol from efflux transporters and promote the inward transport of cells while maintaining their structural integrity (176). Similarly, nanocurcumin has been used in a number of clinical trails and has achieved certain efficacy.

Kaempferol a natural product, is a promising antidiabetic drug. This review provides a systematic summary of the pharmacological mechanisms of kaempferol for the treatment of diabetes. This is a systematic summary of animal and cell experiments. Although this review has some limitations, it adds valuable information that is beneficial in determining new drugs for the treatment of diabetes, from animal research to clinical studies. Large-scale, multicenter, and prospective clinical trials will be conducted in the future to obtain more reliable information.

## Author contributions

YY and ZC conceived the idea and topic for the opinion. YY, XZ and HX collected the data. YY wrote the manuscript. LD, HG and CX reviewed the manuscript and contributed to the intellectual scientific content of the manuscript with domain-specific expertise. All authors contributed to the article and approved the submitted version.

## Funding

This article was funded by the National Natural Science Foundation of China (No. 81774302), the National Natural Science Foundation of China (No. 8197141539), and the Sichuan province of Traditional Chinese medicine academician reserve candidates development project (No. CRS2021067).

## Conflict of interest

The authors declare that the research was conducted in the absence of any commercial or financial relationships that could be construed as a potential conflict of interest.

## Publisher’s note

All claims expressed in this article are solely those of the authors and do not necessarily represent those of their affiliated organizations, or those of the publisher, the editors and the reviewers. Any product that may be evaluated in this article, or claim that may be made by its manufacturer, is not guaranteed or endorsed by the publisher.
